# Feasibility and three months preliminary results of an RCT on the effect of Schroth exercises in adolescent idiopathic scoliosis (AIS)

**DOI:** 10.1186/1748-7161-8-S1-O21

**Published:** 2013-06-03

**Authors:** S Schreiber, EC Parent, DM Hedden, EM Watkins, DL Hill, M Moreau, S Southon, JK Mahood

**Affiliations:** 1 University of Alberta, Faculty of Rehabilitation Medicine, Rehabilitation Science, Edmonton, Canada; 2 University of Alberta, Faculty of Rehabilitation Medicine, Department of Physical Therapy, Edmonton, Canada; 3 University of Alberta, Faculty of Medicine and Dentistry, Department of Surgery, Edmonton, Canada; 4 Glenrose Rehabilitation Hospital, Edmonton, Canada; 5 University of Alberta Hospital, Edmonton, Canada

## Background

Schroth exercises are scoliosis-specific postural exercises aimed at improving appearance, postural control, and prevention of curve progression. An RCT on the effect of Schroth exercises for scoliosis is needed.

## Aim

To determine the recruitment, eligibility, attendance, and compliance rates for an RCT, and estimate the effect size for the selected outcomes.

## Methods

Fifteen patients with AIS, aged 10-18, with curves 10 – 45 degrees, braced or not, were randomized into Schroth, or standard care, groups. The six-month Schroth intervention consists of weekly one-hour long group exercise sessions, combined with a daily 45-minute long home program, consisting of 3-4 exercises prescribed using an algorithm. Compliance was monitored with a logbook. SRS-22r, Spinal Appearance Questionnaire, Sorensen back endurance, and Self-efficacy and Global Rating of Change (GRC) outcomes were measured. The effect sizes of the Schroth exercise treatment on key outcomes were estimated using Cohen’s d (≥0.8=large, 0.5-0.8=moderate, 0.2-0.5=small). Cohen’s d is defined as the difference in change between the group means, Schroth – Standard care, observed from baseline to 3 months, divided by the pooled standard deviation at baseline.

## Results

Of 122 eligible patients, fifteen (12.3%) were enrolled between April and August 2011. Recruitment was 3 per month. Exercise subjects attended 89±9% of the prescribed weekly exercise sessions, and completed 76±8% of the prescribed home exercises. Only one Standard-care subject dropped out due to relocation. For SRS-22r domains, Cohen’s d effect sizes favored standard care: -0.31 for Self-image, -0.72 for Function, and -0.38 for Pain. For SAQ domains, some effect sizes favored standard care: 0.50 for General, 0.46 for Curve, 1.36 for Prominence, 0.57 for Shoulders and 0.35 for Chest. Other SAQ effect sizes favored Schroth: -0.14 for Trunk Shift, -0.31 for waist. The Sorensen test’s effect size was -0.14, and Self-efficacy’s was 0.5, both favoring standard care. The mean difference between groups in GRC was 3.4±1.7 in favor of the Schroth group.

## Conclusion

RCT feasibility was demonstrated by high therapy session attendance and home-exercises compliance. At 3-months, in a small sample, without adjustment for severity, effect sizes favoring Schroth exercises were smaller than anticipated, or favored standard care. We will assess whether our Schroth education, with curve and posture analysis, might have sensitized patients as to how they perceive their scoliosis.
